# Wiskott–Aldrich syndrome and X-linked thrombocytopenia: a review of the clinical and immunological spectrum with a case presentation highlighting glomerulonephritis

**DOI:** 10.3389/fped.2026.1832095

**Published:** 2026-06-04

**Authors:** Ramona Stroescu, Adela Chirita-Emandi, Ruxandra Maria Steflea, Delia Mihailov, Gabriela Doros, Flavia Chisavu, Catalin Munteanu, Mihai Gafencu

**Affiliations:** 1 Department XI -Pediatrics, Victor Babes University of Medicine and Pharmacy, Timisoara, Romania; 2 Spitalul Clinic de Urgenta Pentru Copii Louis Turcanu Timsoara, Timișoara, Romania; 3 Department of Microscopic Morphology, Genetics Discipline, Center for Genomic Medicine, Victor Babes University of Medicine and Pharmacy, Timisoara, Romania; 4 Regional Center of Medical Genetics Timiş, Spitalul Clinic de Urgenta pentru Copii Louis Turcanu Timsoara, Timișoara, Romania

**Keywords:** autoimmunity, children, glomerulonephritis, immunological spectrum, Wiskott–Aldrich

## Abstract

**Background:**

Wiskott–Aldrich syndrome (WAS) is a rare X-linked primary immunodeficiency caused by mutations in the WAS gene, characterized by thrombocytopenia, eczema, recurrent infections, and immune dysregulation. Its milder form, X-linked thrombocytopenia (XLT), exhibits variable autoimmune manifestations. Renal involvement, particularly glomerulonephritis, is uncommon but clinically significant.

**Case presentation:**

We report a 10-year-old boy presenting with severe thrombocytopenia, hyporegenerative anemia, nephritic-range proteinuria, and hematuria, alongside multiple autoantibodies including antiglomerular basement membrane, antineutrophil cytoplasmic antibodies, and antinuclear antibodies. His complement levels were reduced and immunoglobulins elevated. Kidney biopsy revealed IgA nephropathy (Oxford M0 E0 S0 T1 C0) with 30% interstitial fibrosis. Bone marrow studies demonstrated near-complete absence of megakaryocytes. The patient’s family history was notable for thrombocytopenia in the mother and younger brother. Genetic testing confirmed a homozygous c.599+5G>A WAS variant, consistent with XLT.

**Literature review:**

To contextualize these findings, data from the literature, including the IPINet registry (117 patients with WAS/XLT), were reviewed. WAS patients frequently exhibited invasive infections and diverse autoimmune manifestations, including hemolytic anemia, vasculitis, inflammatory bowel disease, arthritis, nephropathy, and celiac disease.

**Discussion:**

Dysregulated T- and B-cell function in WAS/XLT promotes autoantibody formation, contributing to renal injury. IgA nephropathy is the predominant glomerular pathology, mediated by circulating immune complexes containing aberrantly glycosylated IgA1.

**Conclusion:**

This case illustrates the broad spectrum of autoimmunity in WAS/XLT, highlighting the potential for renal involvement. It emphasizes the importance of early recognition, genetic confirmation, and multidisciplinary management. Registry data remain essential for guiding prognosis, monitoring complications, and informing therapeutic strategies, including hematopoietic stem cell transplantation.

## Introduction

Glomerular diseases encompass a broad spectrum of conditions characterized by structural and functional abnormalities of the renal glomerulus. Early identification and age-specific management are vital to prevent chronic kidney disease and end-stage kidney disease (1, 2). Importantly, children are not simply “small adults.” Developmental factors—including immune system maturation, genetic contributions, and environmental exposures—shape disease type and progression (3). Primary glomerular diseases account for most pediatric cases, while adults are more frequently affected by secondary glomerulopathies driven by chronic conditions (e.g., diabetes mellitus, systemic lupus erythematosus, and vasculitis) (4). Despite a lower incidence (children: ∼0.1/100,000/year; adults: ∼0.2–2.5/100,000/year), the burden on childhood quality of life and risk of lifelong complications make early recognition essential (5).

Wiskott–Aldrich syndrome (WAS) is a rare, X-linked recessive primary immunodeficiency caused by variants in the *WAS* gene, primarily affecting males (6). This gene encodes the Wiskott–Aldrich Syndrome protein (WASP), which is crucial for actin cytoskeleton organization in blood cells. Defective WASP leads to impaired immune cell function and platelet formation (7). WAS is characterized by a triad of eczema, thrombocytopenia with small platelets, and recurrent bacterial, viral, and fungal infections, reflecting combined T-cell, B-cell, and sometimes NK-cell dysfunction (8, 9). Additional features include autoimmune disorders (hemolytic anemia, vasculitis, arthritis), increased risk of malignancies (particularly lymphoma), and growth retardation (10).

Although kidney involvement is less common than the classic triad, WAS can be associated with glomerulonephritis in children. The kidney can be affected through autoimmune-mediated glomerular injury. Common types of glomerulonephritis associated with WAS include IgA nephropathy, which often presents with hematuria and mild proteinuria; membranoproliferative glomerulonephritis, which may present with proteinuria, hematuria, or renal impairment; and, rarely, minimal change disease or focal segmental glomerulosclerosis (11–13). Dysregulated T- and B-cell function increases autoantibody production, contributing to immune complex deposition and glomerular inflammation (14, 15).

We aimed to present a rare case of IgA-mediated glomerulonephritis associated with immunodeficiency in relation to WAS and to review the literature to delineate renal involvement in this rare entity.

## Materials and methods

### Case report

Patient evaluation included family and personal history, clinical assessment, genetic testing, laboratory findings, follow-up, and treatment outcomes. The patient underwent genetic testing using next-generation sequencing, in particular whole-exome sequencing. Variant classification followed the ACMG 2015 guidelines (16).

### Literature review

A targeted literature review, including data from the IPINet registry [117 patients with WAS/X-linked thrombocytopenia (XLT)], was conducted in order to better understand the clinical findings observed in patients with WAS. A systematic search was performed using PubMed and Google Scholar, applying combinations of the following keywords: “Wiskott Aldrich,” “autoimmunity,” and “glomerulonephritis.” Boolean operators (“AND,” “OR”) helped refine the search.

Information on the patient's phenotype (WAS/XLT), age at genetic diagnosis, *WAS* variant, autoimmunity status, and autoantibodies (clinical testing) was systematically collected and summarized.

## Results

### Case presentation

A 10-year-old boy was first admitted at age 8 to a regional hospital, where he was diagnosed with idiopathic thrombocytopenic purpura and treated with corticosteroids and immunoglobulins. At discharge, his platelet count had almost normalized. At age 10, the patient developed gastrointestinal symptoms and laterocervical adenitis 1 week before admission. Initially, hospitalization was refused, but 1 week later, biological investigations ordered by the general practitioner revealed acute kidney disease (AKIN III), severe thrombocytopenia (10,000/mm^3^) with normal-size platelets (MPV = 9.5 fL), anemia (Hb = 7.3 g/dL), macroscopic hematuria, and proteinuria (100 mg). The patient was also receiving treatment for a persistent dry cough.

Family history revealed that his mother had thrombocytopenia during pregnancy, with a platelet count of 48,000/mm^3^. Two routine outpatient tests also showed platelet counts below 100,000/mm^3^, while two other tests showed normal values, without any further investigations being performed.

On admission to the nephrology department, the patient presented with severe thrombocytopenia (10,000/mm^3^), hyporegenerative anemia (Hb = 6.9 g/dL, reticulocytes = 0.6%), low C3 and C4 (C3 = 0.74 g/L (N.V. 0.8–1.4), C4 = 0.04 g/L (N.V. 0.14–0.44), elevated IgA, IgM, and IgG [IgG = 27.5 g/L (N.V. 5.4-18.22), IgM = 6.23 g/L (N.V 0.41-1.83), IgA = 4.67 g/L (N.V. 0.21-2.91)], and positive direct and indirect Coombs tests, leading to difficulties in finding a compatible red blood cell unit for transfusion. Diuresis was normal, CRP was elevated (64.5 g/L N.V < 5), and peripheral blood smear confirmed severe thrombocytopenia. Twenty-four-hour urine analysis revealed nephritic-range proteinuria (1,500 mg/24 h) and dysmorphic erythrocytes, with 95% acanthocytes.

**Figure 1:**
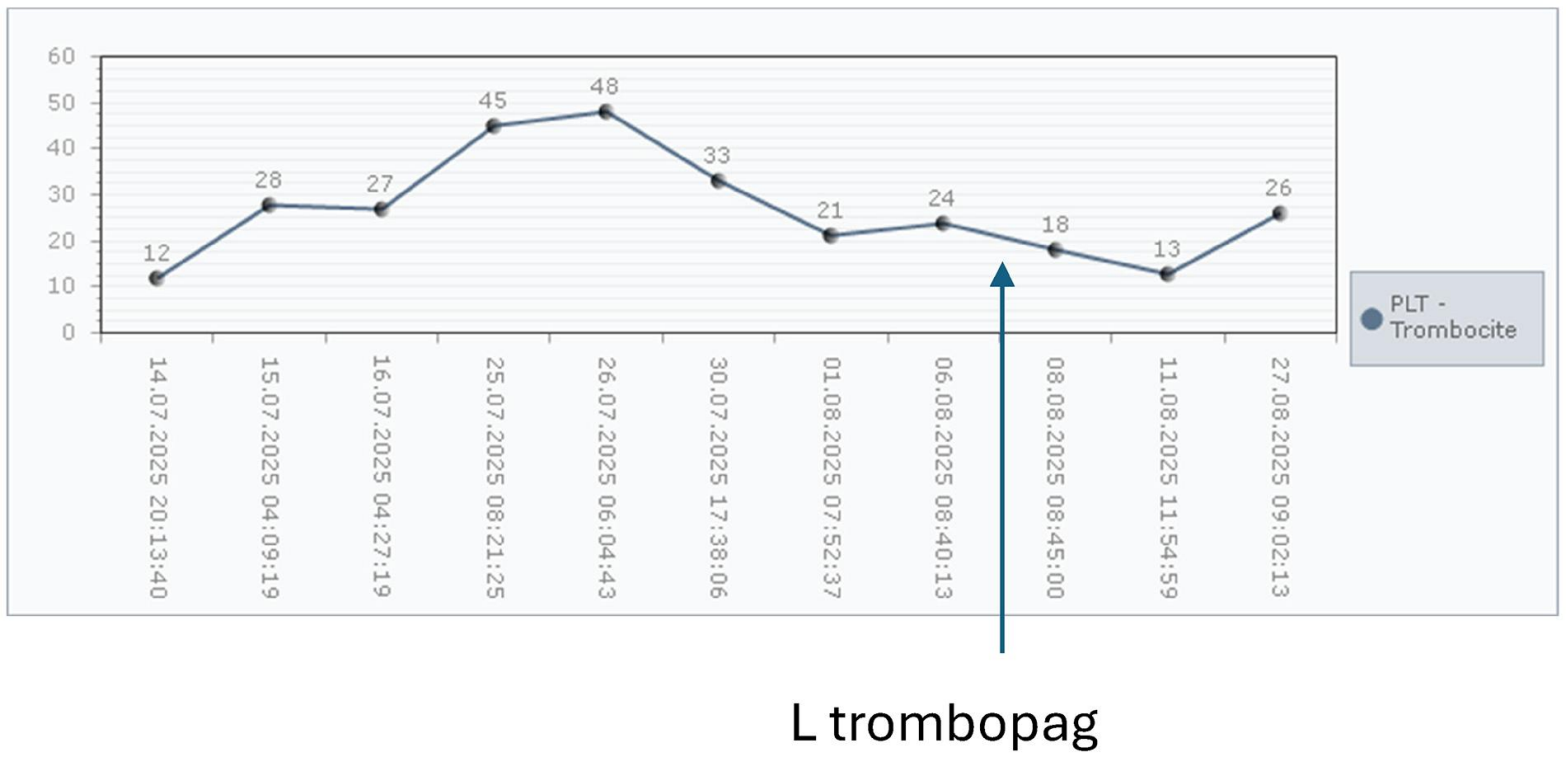
Thrombocyte values.

During the glomerulonephritis work-up, anti-MBG antibodies tested positive, prompting initiation of plasma exchange therapy and corticotherapy 3 pulses. Antineutrophil cytoplasmic antibodies (ANCA) were also positive. However, atypically for ANCA-associated vasculitis, MPO and PR3 were negative, while lactoferrin and bactericidal /permeability increasing protein (BPI) were positive. Therefore, cyclophosphamide pulses were added. Pulmonary CT revealed postero-basal ventilatory disorders. We considered this an overlap and decided to treat for the worst-case scenario. According to the literature, up to one-third of patients with anti-GBM disease may also have ANCA, while up to 5% of patients with ANCA-associated vasculitis may have anti-GBM antibodies. Antinuclear antibodies (ANA), along with antibodies associated with antiphospholipid syndrome, also tested positive. Unusually, these antibodies were anticytoplasmic with a fibrillar/filiform antinuclear pattern. After this, we stopped testing for other antibodies. During the course of the disease, multiple platelet transfusions were required, particularly to perform the kidney biopsy (Figure 1). After 1 month, the hematologist decided to start treatment with Eltrombopag.

Following plasma exchange therapy, the patient’s C3 and C4 levels increased, and IgA, IgM, and IgG normalized.

Two bone marrow aspirations, performed 1 week apart, and one biopsy were performed, revealing an almost complete absence of the megakaryocyte lineage.

**Figure 2:**
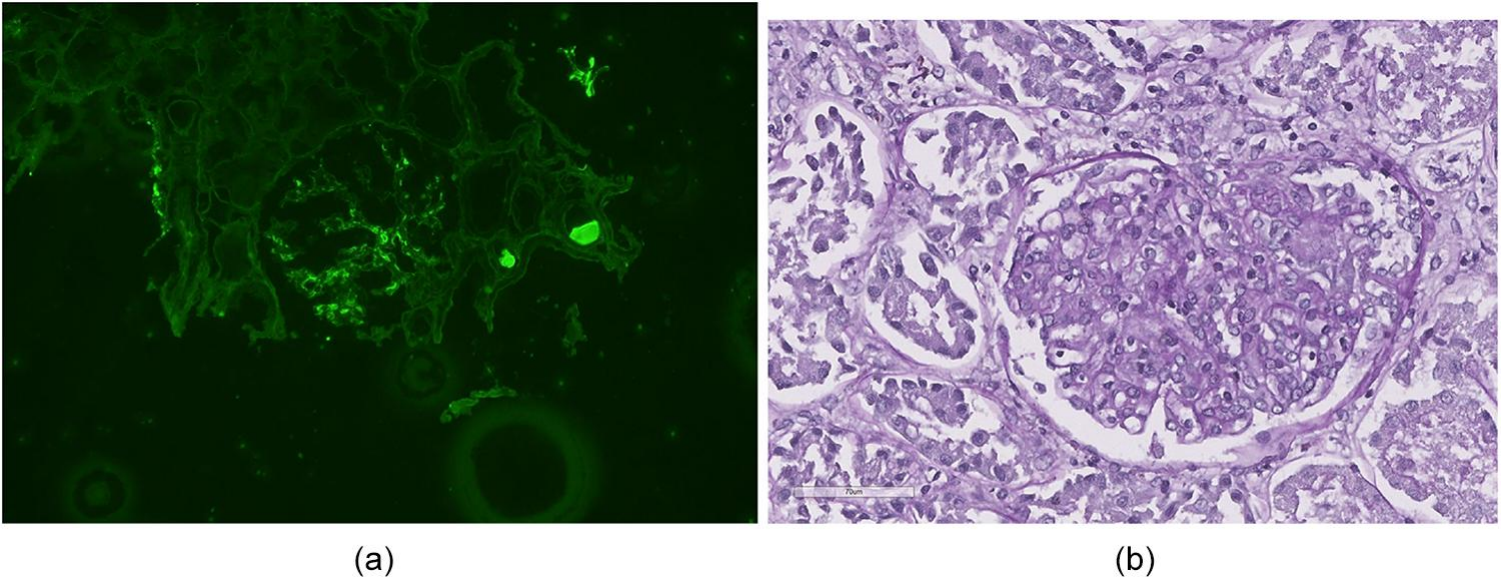
**(a)** Immunofluorescence IgA deposition. **(b)** HE-minimal mesangial matrix expansion.

Subsequent kidney biopsy revealed IgA nephropathy (Oxford M0 E0 S0 T1 C0) without crescent formation, which is atypical for pathogenic anti-glomerular basement membrane (anti-GBM) disease (Figures 2a,b). This discordance suggests that anti-GBM and ANCA positivity in this patient may represent non-pathogenic antibodies related to immune dysregulation rather than true anti-GBM glomerulonephritis. The presence of T1 lesions (30% interstitial fibrosis) indicates significant chronic kidney damage, suggesting that renal involvement may have been present and progressing prior to the acute clinical presentation.

Family history of thrombocytopenia in the patient’s mother (episodes of low thrombocytes, particularly during pregnancy), and younger brother (thrombocytes below 100,000/mm^3^) raised suspicion for Wiskott–Aldrich syndrome-associated IgA nephropathy (immune dysregulation defect).

After 1 month, genetic testing confirmed a hemizygous c.599 + 5G > A WAS variant, consistent with XLT.

The patient is currently being evaluated for a bone marrow transplant. His brother is also being evaluated, with normal kidney function and mild thrombocytopenia.

## Discussion

WAS is an X-linked immunodeficiency characterized by thrombocytopenia and eczema, resulting from mutations in the *WAS* gene. The clinical presentation is variable, with a milder phenotype known as XLT. Autoimmune complications are very common, occurring in 40–70% of patients according to retrospective cohort studies. As advances in infection prevention and treatment have improved survival, managing these autoimmune manifestations has become increasingly important (17–19).

Kidney disease represents a significant complication of WAS and is reported in approximately 3.5–19% of cases (18–20). In some individuals, it can progress to chronic renal failure, ultimately requiring kidney transplantation (21, 22). Despite its clinical relevance, histopathological data remain limited because renal biopsy is often contraindicated due to severe thrombocytopenia. When biopsy findings are available, membranoproliferative glomerulonephritis and interstitial nephritis have occasionally been described, but the most frequently identified pathology is IgA nephropathy (IgAN), which is associated with aberrant IgA glycosylation. IgAN is defined by immune deposits containing a dominant or codominant IgA component within the glomerular mesangium. A key driver of its pathogenesis is the presence of circulating immune complexes (CIC) composed of aberrantly O-glycosylated IgA1. In affected individuals, galactose-deficient IgA1 molecules are produced and subsequently recognized by antiglycan IgG or IgA1 antibodies, leading to CIC formation. These complexes deposit in the renal mesangium and contribute to glomerular inflammation and injury (23–26).

In patients with WAS, enhanced basal activity of β1, 6-N-acetylglucosamine transferase—an enzyme involved in O-linked glycosylation—leads to increased expression of highly O-glycosylated sialophorin, also known as core 2 O-glycans. Because sialophorin plays a crucial role in T-cell activation, its abnormal glycosylation may contribute to the immune dysregulation observed in WAS (27, 28). Notably, studies have demonstrated that retroviral-mediated correction of the *WAS* gene can restore β1, 6-N-acetylglucosamine transferase function and normalize glycosylation profiles in lymphoblastoid WAS B-cells. In WAS, dysregulated T- and B-cell function contributes to excessive autoantibody production (29). This was evident in our patient, who demonstrated elevated immunoglobulin levels along with multiple autoantibodies, including anti-GBM antibodies, ANCA, and ANA.

The presence of elevated immunoglobulin levels along with multiple autoantibodies, including anti-GBM antibodies, ANCA, and ANA in the absence of histological features of crescentic glomerulonephritis further supports the hypothesis that these antibodies may reflect polyclonal B-cell activation and immune dysregulation characteristic of WAS/XLT, rather than being directly pathogenic. Dysregulated T- and B-cell function contributes to excessive autoantibody production. In this context, caution is required when interpreting serological findings, as autoantibody positivity may not always correlate with the underlying renal histopathology.

The two bone marrow aspirations revealing an almost complete absence of the megakaryocyte lineage, combined with normal-sized platelets, is unusual in classical WAS/XLT, where thrombocytopenia is typically associated with decreased platelet size rather than a near absence of megakaryocytes. This observation raises the possibility of an additional mechanism, such as immune-mediated marrow suppression or a more severe hematological phenotype, which warrants further clarification.

The identified variant in our patient, c.599 + 5G > A in the *WAS* gene, is an intronic mutation known to disrupt normal RNA splicing. This alteration is considered a splicing hotspot and is typically associated with the milder clinical phenotype of XLT, as it allows for partial expression of functional WASp. Nonetheless, clinical manifestations can vary even among individuals carrying the same mutation, indicating that additional genetic or environmental modifiers may influence disease severity.

The IPINet registry was established in 1999 under the umbrella of AIEOP (Italian Association of Pediatric Hematology and Oncology) to systematically collect data on patients with primary immunodeficiencies in Italy (29–31). To assess the long-term outcomes of patients with WAS or XLT, individuals with confirmed *WAS* gene mutations have been prospectively enrolled in the WAS/XLT IPINet registry since January 2004. Clinical data are collected at diagnosis and then annually through a centralized web-based platform across participating AIEOP-IPINet centers. For this analysis, data recorded up to December 2018 were retrieved, including a total of 117 patients classified according to Zhu criteria: 92 with WAS and 25 with XLT, all presenting appropriate hematologic abnormalities and a documented *WAS* mutation (31).

### Findings

The median follow-up duration was 6 years (range: 1–30 years), accounting for 1,110 patient-years. At diagnosis, invasive infections—including sepsis, meningitis, brain abscesses, and severe herpesvirus or Candida infections—were reported exclusively in patients with WAS, while none of the XLT patients exhibited invasive infectious disease. Among autoimmune manifestations in WAS, hemolytic anemia was the most frequent (20%), followed by vasculitis (9.3%), inflammatory bowel disease (5%), arthritis (4%), nephropathy (2%), and celiac disease (1%) (29, 30).

### Interpretation

These findings underscore the clinical heterogeneity of WAS and XLT and highlight the need for updated management guidelines tailored to disease severity. Improved tools are required to assess the risk of infectious and autoimmune complications in XLT patients and to evaluate the long-term effects of available treatments, including hematopoietic stem cell transplantation.

Several studies have described a broad spectrum of autoantibodies in Wiskott–Aldrich syndrome and X-linked thrombocytopenia, including cases with multiple autoantibodies and immune-mediated complications, closely resembling the features observed in our patient (32–40).

Table 1 summarizes reported genetic variants, their associated autoimmune manifestations, and detected autoantibodies, alongside the clinical features observed in our patient.

**Table 1:** Results from the literature.

No	Patient's phenotype	Age (years) at report	WAS(NM_000377.3, NP_000368.1): hemizygous variant	ACMG Class	Clinvar ID	Autoimmunity	Autoantibodies (clinical testing)	PMID
1	XLT	22	c.116T > C, p.(Leu39Pro)	LP	NA	vasculitis	ASMA	26409660
2	XLT	8	c.134C > A, p.(Thr45Lys)	LP	4491219	arthritis	ANA, ANCA, ASMA	26409660
3	XLT	15	c.230A > G, p.(Asp77Gly)	LP	2630734	NA	ANA	26409660
4	XLT	11	c.230A > G, p.(Asp77Gly)	LP	2630735	NA	ANA	26409660
5	WAS	2	c.190T > C, p.(Trp64Arg)	P	1686299	NA	ANA	26409660
6	WAS	5	c.1085dup, p.(Gly363ArgfsTer132)	LP	NA	NA	Plt, TPO	26409660
7	WAS	3	c.190T > C, p.(Trp64Arg)	P	1686299	AIHA, IBD	Coombs	26409660
8	WAS	5	c.190T > C, p.(Trp64Arg)	P	1686299	AIHA, IBD, vasculitis	Coombs	26409660
9	WAS	3	NA (p. V106Cfs*15)	NA	NA	vasculitis	PL, Plt	26409660
10	WAS	9	NA (p. E67Efs*4)	NA	NA	IBD	NA	26409660
11	WAS	1.6	NA (p. D495Mfs*98)	NA	NA	NA	Coombs, ANCA	26409660
12	WAS	0.8	c.257G > A, p.(Arg86His)	P	11115	NA	ANA, Coombs, Plt	26409660
13	WAS	2	c.559 + 5G > A, p-	P	372545	NA	TPO	26409660
14	WAS	3	c.559 + 5G > A, p-	P	372545	NA	TPO	26409660
15	WAS	48	c.559 + 5G > A, p-	P	372545	NA	PL	26409660
16	XLT	10	c.223G > A, p.(Val75Met)	P	265289	IgA nephropathy	No	34758123
17	WAS	0.2	c.1240_1247del, p.(P414Sfs*78)	P	NA	IgA	No	34758123
18	WAS	0.7	c.221T > C, p.(Phe74Ser)	LP	NA	IBD	No	34758123
19	WAS	NA	NA (IVS6 + 1)	NA	NA	Membranoproliferative glomerulonephritis associated with anti-glomerular basement-membrane antibody	Anti MBG	21067383
20	WAS	10	c.1020_1047del, p.(Leu342HisfsTer94)			Ig A nephropathy	NA	20232122
21	WAS	3	c.143C > T, p.(Thr48Ile)	LP	NA	HSP; ulcerative colitis; leukocytoclastic vasculitis; amyloidosis; AIT; HSP with IgA nephropathy	NA	32812413
22	WAS	12.3	NA [nv(X) (5,721, 11,840)]	NA	NA	Recurrent arthritis; vasculitis, Henoch–Schönlein purpura with nephritic-nephrotic syndrome; panuveitis; Crohn's-like enterocolitis; perianal fistulae and abscesses; pyoderma gangrenosum	NA	30981783
23	WAS	1.7	c.1509A > T, (Ter503CysextTer79)	LP	NA	Colitis or gastrointestinal bleeding, mucosal bleeding, suspected food allergy	NA	30981783
24	WAS	1.7	NA [Exon 10, 1595del, proximal breakpoint (5247_6842del) in genomic DNA]	NA	NA	Food allergy, hepatomegaly, splenomegaly, inflammatory lymphadenopathy; eosinophilia	NA	30981783
25	WAS	23	NA (Exon 6, p.L179fs260*)	NA	NA	Vasculitis; arthritis; IgA nephropathy	NA	17296785
26	WAS	1.8	NA (Exon 4 431G > A)	NA	NA	Colitis, vasculitis	NA	17296785
27	WAS	NA	NA (Exon 10, p.K336*)	NA	NA	Glomerulonephritis	NA	26961359
28	WAS	10	c.559 + 5G > A, p-	P	372545	IgA nephropathy	ANA, ANCA, anti MBG Coombs	This study

NA, not available; AIHA, autoimmune hemolytic anemia; ANA, antinuclear antibodies; ANCA, antineutrophil cytoplasmic antibodies; ASMA, antismooth muscle antibodies; IBD, inflammatory bowel disease; PL, antiphospholipid antibodies; Plt, anti-platelet antibodies; TPO, anti-thyroid peroxidase antibodies.

The table provides a comprehensive overview of previously reported patients with WAS and XLT, detailing age at presentation, specific genetic variants, associated autoimmune manifestations, and the results of autoantibody testing. Data are drawn from multiple published studies, including Crestani, Jiali Jang, Botzug, Lee, Haskologlu, Ferrua, Marangoni, and Shigemura, and are presented alongside the features observed in our patient.

The reported cases illustrate the broad clinical heterogeneity of WAS and XLT. Patients with XLT generally present milder disease but can still exhibit autoimmune manifestations such as vasculitis, arthritis, and IgA nephropathy. WAS patients show a wider spectrum of complications, ranging from autoimmune cytopenias (e.g., autoimmune hemolytic anemia, thrombocytopenia) to systemic autoimmune disorders including inflammatory bowel disease, vasculitis, Henoch–Schönlein purpura, panuveitis, Crohn's-like enterocolitis, pyoderma gangrenosum, and IgA nephropathy.

Autoantibody testing demonstrates considerable variability, reflecting the underlying immune dysregulation. Detected antibodies include ANA, ANCA, anti-glomerular basement membrane antibodies, antismooth muscle antibodies (ASMA), Coombs positivity, platelet-specific antibodies, and thyroid autoantibodies. Some patients present with multiple autoantibodies and overlapping autoimmune disorders, highlighting the complexity of immune dysregulation in WAS/XLT.

Our patient's features, including the presence of multiple autoantibodies and autoimmune manifestations, mirror those observed in previously reported cases, underscoring the recurrent patterns of autoimmunity associated with specific WAS gene variants. This compilation emphasizes the importance of systematic evaluation for autoimmune complications in both WAS and XLT and supports the role of genotype–phenotype correlations in anticipating clinical outcomes.

## Conclusions

Pediatric glomerular diseases require distinct diagnostic and therapeutic approaches. Renal biopsy and genetic testing are increasingly crucial in guiding treatment. Advances in immunotherapies and precision medicine promise better long-term kidney outcomes. Understanding how glomerulopathies differ across age groups supports timely intervention, improved survival, and reduced lifelong disease burden. WAS and XLT are associated with a wide spectrum of autoimmune manifestations and diverse autoantibody profiles. While XLT generally presents with milder disease, patients may still develop significant autoimmune complications. WAS patients exhibit broader immune dysregulation, frequently involving multiple organ systems and hematologic autoimmunity. The presence of multiple autoantibodies in many cases—including our patient—highlights the complexity of immune dysregulation and the importance of systematic monitoring. These findings underscore the need for genotype-informed risk assessment and individualized management strategies to anticipate and address autoimmune complications in both WAS and XLT.

## Data Availability

The raw data supporting the conclusions of this article will be made available by the authors, upon request.
